# Stability Analysis of Nonlinear Systems with Slope Restricted Nonlinearities

**DOI:** 10.1155/2014/278305

**Published:** 2014-01-28

**Authors:** Xian Liu, Jiajia Du, Qing Gao

**Affiliations:** Key Lab of Industrial Computer Control Engineering of Hebei Province, Institute of Electrical Engineering, Yanshan University, Qinhuangdao 066004, China

## Abstract

The problem of absolute stability of Lur'e systems with sector and slope restricted nonlinearities is revisited. Novel time-domain and frequency-domain criteria are established by using the Lyapunov method and the well-known Kalman-Yakubovich-Popov (KYP) lemma. The criteria strengthen some existing results. Simulations are given to illustrate the efficiency of the results.

## 1. Introduction

Absolute stability of nonlinear systems has been investigated comprehensively for the past several decades [1–12]. It is well known that the Popov criterion and the circle criterion are two classical results with the forms of frequency-domain inequalities (FDIs), which are turned out to be equivalent to some linear matrix inequalities (LMIs). This not only gives the opportunity to use the powerful LMI toolbox [13] to study absolute stability, but also gives the opportunity to consider the controller design problems. In [14], absolute stability of single-input and single-output Lur'e systems with a sector and slope restricted nonlinearity is brought forward. It is pointed out that the slope restriction on the nonlinearity strengthens the Popov criterion by adding an additional term to the original FDI of the criterion. Much work [15–22] on the slope restricted and multivariable problem has been done by using a Lur'e-Postnikov function or an extended Lur'e-Postnikov function.

In this paper, both time-domain criterion and frequency-domain criterion for absolute stability of Lur'e systems with sector and slope restricted nonlinearities are presented based on the Lyapunov method and the KYP lemma. Some mathematical tools are used through the derivation of the absolute stability criterion. Compared with some existing results, the proposed results are less conservative. This should be owed to the effect of the slope restricted conditions on the nonlinearities. The rest of the paper is organized as follows. In Section 2, the system description and some preliminaries are presented. Time-domain and frequency-domain criteria for absolute stability of the system are given in Section 3. Numerical examples are given in Section 4 and some concluding remarks are given in Section 5.

Throughout this paper, the superscript ∗ means transpose of real matrices and conjugate transpose of complex matrices. For a Hermitian matrix *W*, *W* > 0 (*W* ≥ 0) denotes that *W* is a positive definite (semidefinite) matrix and *W* < 0 denotes that *W* is a negative definite matrix. *Re*{*Y*} means (1/2)(*Y* + Y*) for any real or complex square matrix *Y*.

## 2. Problem Statement

Consider the following multi-input and multioutput Lur'e system

(1) $$\begin{array}{c} \dot{x}\left(t\right)=Ax\left(t\right)+B\varphi \left(\sigma \left(t\right)\right),\\ \sigma \left(t\right)=C^{*}x\left(t\right), \end{array}$$

where *A* ∈ ℝ^*n*×*n*^, *B* ∈ ℝ^*n*×*m*^, and *C* ∈ ℝ^*n*×*m*^ are real matrices, *φ*(0) = 0, $\sigma (t)=\left[\begin{array}{c} \sigma_{1}(t)\\ \vdots \\ \sigma_{m}(t) \end{array}\right]$ is the output, $\varphi (\sigma (t))=\left[\begin{array}{c} \varphi_{1}(\sigma_{1}(t))\\ \vdots \\ \varphi_{m}(\sigma_{m}(t)) \end{array}\right]$ is piecewise continuously differentiable on ℝ^*m*^, and *φ*_*i*_(*σ*_*i*_(*t*))  (*i* = 1,2,…, *m*) are assumed to satisfy

(2) $$\begin{array}{c} \gamma_{1i}\sigma_{i}^{2}\left(t\right)\leq \varphi_{i}\left(\sigma_{i}\left(t\right)\right)\sigma_{i}\left(t\right)\leq \delta_{1i}\sigma_{i}^{2}\left(t\right), \end{array}$$

(3) $$\begin{array}{c} \gamma_{2i}\leq \frac{d\varphi_{i}\left(\sigma_{i}\left(t\right)\right)}{d\sigma_{i}\left(t\right)}\leq \delta_{2i}, \end{array}$$

where *γ*_2*i*_ ≤ *γ*_1*i*_, *δ*_2*i*_ ≥ *δ*_1*i*_, *γ*_2*i*_ ≤ 0, and *δ*_2*i*_ ≥ 0. The inequalities (2) and (3) denote sector restriction and slope restriction on *φ*(*σ*(*t*)), respectively. Let Γ_1_ = diag⁡(*γ*_11_,…, *γ*_1*m*_), Δ_1_ = diag⁡(*δ*_11_,…, *δ*_1*m*_), Γ_2_ = diag⁡(*γ*_21_,…, *γ*_2*m*_), Δ_2_ = diag⁡(*δ*_21_,…, *δ*_2*m*_). Then Γ_2_ − Γ_1_ ≤ 0, Δ_2_ − Δ_1_ ≥ 0, Γ_2_ ≤ 0, and Δ_2_ ≥ 0. Setting *ψ*_*i*_(*σ*_*i*_(*t*)) = *dφ*_*i*_(*σ*_*i*_(*t*))/*dt*, (3) is formulated as follows:

(4) $$\begin{array}{c} \gamma_{2i}\leq \frac{\psi_{i}\left(\sigma_{i}\left(t\right)\right)}{{\dot{\sigma }}_{i}\left(t\right)}\leq \delta_{2i}. \end{array}$$

The transfer function from *φ*(*σ*(*t*)) to −*σ*(*t*) is denoted as *χ*(*s*) = C*(*A* − *sI*)^−1^ *B*.

System (1) is called to be absolutely stable if the equilibrium point *x*(*t*) = 0 is globally asymptotically stable for all nonlinear vector valued functions  *φ*(*σ*(*t*)) satisfying (2) and (3). In the following sections, less conservative absolute stability criteria including time-domain criterion and frequency-domain criterion for system (1) are given. Before studying these problems, first we introduce the KYP lemma and Schur complement. These lemmas will be used repeatedly in this paper to get our main results.

**Lemma 1 (KYP lemma [23])**

Given that *A* ∈ ℝ^*n*×*n*^, *B* ∈ ℝ^*n*×*m*^, and symmetric matrix Σ ∈ ℝ^(*n*+*m*)×(*n*+*m*)^, with det⁡(*jωI* − *A*) ≠ 0 for *ω* ∈ ℝ, and the pair (*A*, *B*) is controllable, the following two statements are equivalent.

- ${\left[\begin{array}{c} {\left(j\omega I-A\right)}^{-1}B\\ I \end{array}\right]}^{*}\Sigma \left[\begin{array}{c} {\left(j\omega I-A\right)}^{-1}B\\ I \end{array}\right]\leq 0$,  for all *ω* ∈ ℝ.
- There exists a matrix *P* = P* such that $\left[\begin{array}{cc} A^{*}P+PA & PB\\ B^{*}P & 0 \end{array}\right]+\Sigma \leq 0$. The equivalence for strict inequalities holds even if (*A*, *B*) is not controllable.

**Lemma 2 (Schur complement [24])**

The LMI $\left[\begin{array}{cc} S_{11} & S_{12}\\ S_{12}^{*} & -S_{22} \end{array}\right]<0$ is equivalent to one of the following statements:

- *S*_22_ > 0 and *S*_11_ + *S*_12_ *S*_22_^−1^ *S*_12_* < 0;
- *S*_11_ < 0 and *S*_22_ + *S*_12_*S_11_^−1^ *S*_12_ > 0.

## 3. Main Results

We choose the following Lur'e-Postnikov function:

(5) $$\begin{array}{c} V\left(x\left(t\right)\right)=x^{*}\left(t\right)Px\left(t\right)+\overset{m}{\underset{i=1}{\sum }}\lambda_{i}\overset{\sigma_{i}(t)}{\underset{0}{\int }}\varphi_{i}\left(s\right)ds \end{array}$$

as the Lyapunov function, where *P* = P* and *λ*_*i*_ ∈ ℝ  (*i* = 1,2,…, *m*) are necessary to be determined. It should be pointed out that *P* is not necessary to be positive definite and *λ*_*i*_  (*i* = 1,2,…, *m*) are not necessary to be nonnegative.

**Theorem 3**

System (1) is absolutely stable for all *φ*(*σ*(*t*)) satisfying (2) and (3) if *A* + *B*Γ_1_ C* is Hurwitzian and there exist diagonal matrices Λ = diag⁡(*λ*_1_,…, *λ*_*m*_), *T*_1_ ≥ 0, *T*_2_ > 0, and symmetric matrices *P* such that the LMI is feasible:

(6) $$\begin{array}{c} \left[\begin{array}{ccc} A^{*}P+PA+\Sigma_{11} & PB+\Sigma_{12} & \Sigma_{13}\\ B^{*}P+\Sigma_{12}^{*} & \Sigma_{22} & \Sigma_{23}\\ \Sigma_{13}^{*} & \Sigma_{23}^{*} & -T_{2} \end{array}\right]<0, \end{array}$$

where

(7) $$\begin{array}{c} \Sigma_{11}=-C\Gamma_{1}T_{1}\Delta_{1}C^{*}-A^{*}C\Gamma_{2}T_{2}\Delta_{2}C^{*}A,\\ \Sigma_{12}=\frac{1}{2}A^{*}C\Lambda +\frac{1}{2}CT_{1}\left(\Gamma_{1}+\Delta_{1}\right)-A^{*}C\Gamma_{2}T_{2}\Delta_{2}C^{*}B,\\ \Sigma_{13}=\frac{1}{2}A^{*}CT_{2}\left(\Gamma_{2}+\Delta_{2}\right),\;\;\Sigma_{23}=\frac{1}{2}B^{*}CT_{2}\left(\Gamma_{2}+\Delta_{2}\right),\\ \Sigma_{22}=\frac{1}{2}\Lambda C^{*}B+\frac{1}{2}B^{*}C\Lambda -T_{1}-B^{*}C\Gamma_{2}T_{2}\Delta_{2}C^{*}B. \end{array}$$

**Proof**

We will demonstrate that the given conditions imply the negative definiteness of $\dot{V}(x(t))$ and the positive definiteness of *V*(*x*(*t*)).

Taking the derivative of *V*(*x*(*t*)) along the trajectory of (1), we have

(8) $$\begin{array}{c} \dot{V}\left(x\left(t\right)\right)=x^{*}\left(t\right)P\dot{x}\left(t\right)+{\dot{x}}^{*}\left(t\right)Px\left(t\right)+\varphi^{*}\left(\sigma \left(t\right)\right)\Lambda C^{*}\dot{x}\left(t\right). \end{array}$$

Conditions (2) and (4) for *φ*_*i*_(*σ*_*i*_(*t*)) are equivalent to

(9) $$\begin{array}{c} u_{1i}\left(x_{i}\right)=\left(\varphi_{i}\left(\sigma_{i}\left(t\right)\right)-\gamma_{1i}\sigma_{i}\left(t\right)\right)\left(\varphi_{i}\left(\sigma_{i}\left(t\right)\right)-\delta_{1i}\sigma_{i}\left(t\right)\right)\leq 0,\\ u_{2i}\left(x_{i}\right)=\left(\psi_{i}\left(\sigma_{i}\left(t\right)\right)-\gamma_{2i}{\dot{\sigma }}_{i}\left(t\right)\right)\left(\psi_{i}\left(\sigma_{i}\left(t\right)\right)-\delta_{2i}{\dot{\sigma }}_{i}\left(t\right)\right)\leq 0. \end{array}$$

For any *t*_1*i*_ ≥ 0 and *t*_2*i*_ > 0, *i* = 1,2,…, *m*, it follows

(10) ∑i=1mt1iu1i(xi)=φ∗(σ(t))T1φ(σ(t)) −12φ∗(σ(t))(Γ1+Δ1)T1C∗x −12x∗CT1(Γ1+Δ1)φ(σ(t)) +x∗CΓ1T1Δ1C∗x≤0,∑i=1mt2iu2i(xi)=ψ∗(σ(t))T2ψ(σ(t)) −12ψ∗(σ(t))(Γ2+Δ2)T2C∗x˙ −12x˙∗CT2(Γ2+Δ2)ψ(σ(t)) +x˙∗CΓ2T2Δ2C∗x˙≤0,

where *T*_1_ = diag⁡(*t*_11_,…, *t*_1*m*_) ≥ 0 and *T*_2_ = diag⁡(*t*_21_,…, *t*_2*m*_) > 0. Then

(11) V˙(x(t))≤x∗(t)Px˙(t)+x˙∗(t)Px(t)+φ∗(σ(t))ΛC∗x˙(t) −∑i=1mt1iu1i(xi)−∑i=1mt2iu2i(xi).

The given condition (6) guarantees the negative definiteness of the right hand of (11). Consequently, $\dot{V}(x(t))$ is negative definite.

Now we are only left to demonstrate that *V*(*x*(*t*)) is positive definite. In (5), *P* is only a symmetric matrix but not a positive definite matrix and *λ*_*i*_ may be a positive or negative number. Therefore, the proof of the positive definiteness of *V*(*x*(*t*)) is a little difficult and complex. Without loss of generality, letting *λ*_*i*_ < 0  (*i* = 1,2,…, *k*) and *λ*_*i*_ ≥ 0  (*i* = *k* + 1,…, *m*)  (0 ≤ *k* ≤ *m*), then *V*(*x*(*t*)) has the following form:

(12) V(x(t))=x∗(t)Px(t)+∑i=1mλi∫0σi(t)(γ1is+φi(s)−γ1is) ds≥x∗(t)[P+12CΛΓ1C∗+12CΛ(Δ1k−Γ1k)C∗]x(t) +∑i=k+1mλi∫0σi(t)(φi(s)−γ1is) ds,

where Δ_1*k*_ = diag⁡(*δ*_11_,…, *δ*_1*k*_, 0,…, 0) and Γ_1*k*_ = diag⁡(*γ*_11_,…, *γ*_1*k*_, 0,…, 0). Since (2) implies *σ*_*i*_(*t*)(*φ*_*i*_(*σ*_*i*_(*t*)) − *γ*_1*i*_ *σ*_*i*_(*t*)) ≥ 0, ∑_*i*=*k*+1_^*m*^ *λ*_*i*_∫_0_^*σ*_*i*_(*t*)^(*φ*_*i*_(*s*) − *γ*_1*i*_ *s*) *ds* ≥ 0 is satisfied. Then *V*(*x*(*t*)) is positive definite if *P* + (1/2)*C*ΛΓ_1_ C* + (1/2)*C*Λ(Δ_1*k*_ − Γ_1*k*_)C* is positive definite, which is proved in what follows.

Denote *A*_1_ = *A* + *B*Γ_1_ C*, *P*_1_ = *P* + (1/2)*C*ΛΓ_1_ C*, *A*_*k*_ = *A*_1_ + *B*(Δ_1*k*_ − Γ_1*k*_)C*, and *P*_*k*_ = *P*_1_ + (1/2)*C*Λ(Δ_1*k*_ − Γ_1*k*_)C*. Firstly, the given conditions imply that $A+B\Gamma_{1}C^{*}+B\tilde{\Delta }C^{*}$ is Hurwitzian for any diagonal matrix $\tilde{\Delta }$ satisfying $0\leq \tilde{\Delta }\leq \Delta_{1}-\Gamma_{1}$. Actually, the matrix $A+B\Gamma_{1}C^{*}+B\tilde{\Delta }C^{*}$ is Hurwitzian for $\tilde{\Delta }=0$ in virtue of the given conditions. So we will demonstrate that $A+B\Gamma_{1}C^{*}+B\tilde{\Delta }C^{*}$ is Hurwitzian for any diagonal matrix $\tilde{\Delta }$ satisfying $0<\tilde{\Delta }\leq \Delta_{1}-\Gamma_{1}$. We assume there exists a diagonal matrix $\tilde{\Delta }$ satisfying $0<\tilde{\Delta }\leq \Delta_{1}-\Gamma_{1}$ such that the matrix $A+B\Gamma_{1}C^{*}+B\tilde{\Delta }C^{*}=A_{1}+B\tilde{\Delta }C^{*}$ is not Hurwitzian. On the one hand, a number *α* satisfying 0 < *α* ≤ 1 can be found such that

(13) det⁡(jω0I−A1−αBΔ~C∗) =det⁡(jω0I−A1)det⁡(I−αC∗(jω0I−A1)−1BΔ~)=0

holds for certain *ω*_0_ ∈ ℝ. Since *A*_1_ is Hurwitzian, det⁡(*jω*_0_ *I* − *A*_1_) ≠ 0 and $\det(I-\alpha C^{*}{\left(j\omega_{0}I-A_{1}\right)}^{-1}B\tilde{\Delta })=0$ are followed. The latter formula indicates that there exists a vector *ν* ≠ 0 such that

(14) $$\begin{array}{c} \nu^{*}\left(I-\alpha \tilde{\Delta }G^{*}\left(j\omega_{0}\right)\right)=0, \end{array}$$

where $\nu^{*}\tilde{\Delta }\neq 0$ and *G*(*jω*_0_) = C*(*jω*_0_ *I* − *A*_1_)^−1^ *B*. Then we derive

(15) ν∗Δ~{−T1+12(Δ1−Γ1)T1G(jω0)+12G∗(jω0)T1(Δ1−Γ1)+12jω0ΛG(jω0)+12[jω0ΛG(jω0)]∗− ω02G∗(jω0)Γ2T2Δ2G(jω0)}Δ~ν≥0.

On the another hand, pre- and postmultiplying both sides of (6) by $W_{1}=\left[\begin{array}{ccc} I & C\Gamma_{1} & 0\\ 0 & I & 0\\ 0 & 0 & I \end{array}\right]$ and *W*_1_*, we have

(16) $$\begin{array}{c} \left[\begin{array}{ccc} A_{1}^{*}P_{1}+P_{1}A_{1}-A_{1}^{*}C\Gamma_{2}T_{2}\Delta_{2}C^{*}A_{1} & {\overset{-}{\Sigma }}_{12} & {\overset{-}{\Sigma }}_{13}\\ {\overset{-}{\Sigma }}_{12}^{*} & \Sigma_{22} & \Sigma_{23}\\ {\overset{-}{\Sigma }}_{13}^{*} & \Sigma_{23}^{*} & -T_{2} \end{array}\right]<0, \end{array}$$

where

(17) Σ−12=P1B+12A1∗CΛ+12CT1(Δ1−Γ1)−A1∗CΓ2T2Δ2C∗B,Σ−13=12A1∗CT2(Γ2+Δ2).

By the Schur complement, (16) implies

(18) $$\begin{array}{c} \left[\begin{array}{cc} A_{1}^{*}P_{1}+P_{1}A_{1}-A_{1}^{*}C\Gamma_{2}T_{2}\Delta_{2}C^{*}A_{1} & {\overset{-}{\Sigma }}_{12}\\ {\overset{-}{\Sigma }}_{12}^{*} & \Sigma_{22} \end{array}\right]<0. \end{array}$$

From the KYP lemma, we derive that (18) holds if and only if

(19) [(jωI−A1)−1BI]∗[Σ^11Σ^12Σ^12∗Σ22][(jωI−A1)−1BI]<0,∀ω∈ℝ,

where ${\hat{\Sigma }}_{11}=-A_{1}^{*}C\Gamma_{2}T_{2}\Delta_{2}C^{*}A_{1}$ and ${\hat{\Sigma }}_{12}=\left(1/2\right)A_{1}^{*}C\Lambda +\left(1/2\right)CT_{1}(\Delta_{1}-\Gamma_{1})-A_{1}^{*}C\Gamma_{2}T_{2}\Delta_{2}C^{*}B$. Inequality (19) is equivalent to

(20) −T1+12(Δ1−Γ1)T1G(jω)+12G∗(jω)T1(Δ1−Γ1)   +12jωΛG(jω)+12[jωΛG(jω)]∗   −ω2G∗(jω)Γ2T2Δ2G(jω)<0, ∀ω∈ℝ

in terms of the equalities *G*(*jω*) = C*(*jωI* − *A*_1_)^−1^ *B* and *jω* *G*(*jω*) = C*A_1_(*jωI* − *A*_1_)^−1^ *B* + C*B. Letting *ω* = *ω*_0_ in (20) and pre- and postmultiplying both sides of the resulting inequality by $\nu^{*}\tilde{\Delta }$ and $\tilde{\Delta }\nu$, it follows that

(21) ν∗Δ~{−T1+12(Δ1−Γ1)T1G(jω0)+12G∗(jω0)T1(Δ1−Γ1)+12jω0Λ×G(jω0)+12[jω0ΛG(jω0)]∗− ω02G∗(jω0)Γ2T2Δ2G(jω0)}Δ~ν =ν∗Δ~T1[1α(Δ1−Γ1)−Δ~]ν+ω02α2ν∗(−Γ2T2Δ2)ν<0.

We can observe that (15) and (21) are contradictive, which means that the assumption is not true and $A+B\Gamma_{1}C^{*}+B\tilde{\Delta }C^{*}$ is Hurwitzian for any diagonal matrix $\tilde{\Delta }$ satisfying $0\leq \tilde{\Delta }\leq \Delta_{1}-\Gamma_{1}$. Therefore, the matrix *A*_*k*_ = *A* + *B*Γ_1_ C* + *B*(Δ_1*k*_ − Γ_1*k*_)C* is Hurwitzian. Secondly, the given conditions imply that *P* + (1/2)*C*ΛΓ_1_ C* + (1/2)*C*Λ(Δ_1*k*_ − Γ_1*k*_)C* is positive definite. Actually, pre- and postmultiplying both sides of (16) by $W_{2}=\left[\begin{array}{ccc} I & C(\Delta_{1k}-\Gamma_{1k}) & 0\\ 0 & I & 0\\ 0 & 0 & I \end{array}\right]$ and *W*_2_* yield

(22) $$\begin{array}{c} \left[\begin{array}{ccc} \Xi_{11} & \Xi_{12} & \Xi_{13}\\ \Xi_{12}^{*} & \Sigma_{22} & \Sigma_{23}\\ \Xi_{13}^{*} & \Sigma_{23}^{*} & -T_{2} \end{array}\right]<0, \end{array}$$

where

(23) Ξ11=Ak∗Pk+PkAk+C(Δ1k−Γ1k) ×T1[(Δ1−Γ1)−(Δ1k−Γ1k)]C∗ −Ak∗CΓ2T2Δ2C∗Ak,Ξ12=PkB+12Ak∗CΛ−Ak∗CΓ2T2Δ2C∗B +12CT1(Δ1−Γ1)−CT1(Δ1k−Γ1k),Ξ13=12Ak∗CT2(Γ2+Δ2).

Inequality (22) implies *Ξ*_11_ < 0. According to 0 ≤ Δ_*k*_ − Γ_*k*_ ≤ Δ_1_ − Γ_1_, *T*_2_ > 0, Γ_2_ ≤ 0, Δ_2_ ≥ 0, *A*_k_*P_*k*_ + *P*_*k*_ *A*_*k*_ < 0 is followed. The matrix *A*_*k*_ is Hurwitzian, which results in the positive definiteness of *P*_*k*_ and *V*(*x*(*t*)). This completes the proof.

It is found in the proof of Theorem 3, more exactly in inequality (16), that if (6) holds, then *A* + *B*Γ_1_ C* is Hurwitzian if and only if *P* + (1/2)*C*ΛΓ_1_ C* > 0.

**Theorem 4**

System (1) is absolutely stable for all *φ*(*σ*(*t*)) satisfying (2) and (3) if there exist diagonal matrices Λ, *T*_1_ ≥ 0, *T*_2_ > 0, symmetric matrices *P*, *Q* > 0 such that *P* + (1/2)*C*ΛΓ_1_ C* > 0 and the LMI (6) holds.

**Remark 5**

Theorem 3 is derived directly by using the time-domain method and can be used to study multi-input and multioutput Lur'e systems. Inequality (6) in Theorem 3 is in the form of LMI, which is easier to be solved by means of the LMI toolbox.

The LMI (6) can be transformed into an equivalent FDI. Thus, a frequency-domain criterion for (1) is given as follows.

**Theorem 6**

System (1) is absolutely stable for all *φ*(*σ*(*t*)) satisfying (2) and (3) if the matrix *A* + *B*Γ_1_ C* is Hurwitzian and there exist diagonal matrices Λ, *T*_1_ ≥ 0, *T*_2_ > 0 such that the following frequency-domain inequality holds

(24) Re{[I+Γ1χ(jω)]∗T1[I+Δ1χ(jω)]+jωΛχ(jω)+ω2[I+Γ2χ(jω)]∗T2[I+Δ2χ(jω)]}>0,ω∈ℝ.

**Proof**

Let $\overset{-}{P}=\left[\begin{array}{cc} P & 0\\ 0 & 0 \end{array}\right]$, $\overset{-}{A}=\left[\begin{array}{cc} A & B\\ 0 & 0 \end{array}\right]$, and $L=\left[\begin{array}{c} 0\\ I \end{array}\right]$. Inequality (6) can be rewritten as

(25) $$\begin{array}{c} \left[\begin{array}{cc} \overset{-}{P}\;\overset{-}{A}+{\overset{-}{A}}^{*}\overset{-}{P}+\Omega_{11} & \overset{-}{P}L+\Omega_{12}\\ L^{*}\overset{-}{P}+\Omega_{12}^{*} & -T_{2} \end{array}\right]<0, \end{array}$$

where

(26) $$\begin{array}{c} \Omega_{11}=\left[\begin{array}{cc} \Sigma_{11} & \Sigma_{12}\\ \Sigma_{12}^{*} & \Sigma_{22} \end{array}\right],\;\;\Omega_{12}=\left[\begin{array}{c} \Sigma_{13}\\ \Sigma_{23} \end{array}\right]. \end{array}$$

According to the KYP lemma, (25) is equivalent to

(27) [(jωI−A−)−1LI]∗[Ω11Ω12Ω12∗−T2][(jωI−A−)−1LI]<0,∀ω∈ℝ.

By simple computations, we have

(28) $$\begin{array}{c} {\left(j\omega I-\overset{-}{A}\right)}^{-1}L=\frac{1}{j\omega }\left[\begin{array}{c} {\left(j\omega I-A\right)}^{-1}B\\ I \end{array}\right],\\ C^{*}A{\left(A-j\omega I\right)}^{-1}B=C^{*}B+j\omega \chi \left(j\omega \right), \end{array}$$

where *χ*(*jω*) = C*(*A*−*jωI*)^−1^ *B*. Substituting (28) into (27), the equivalence between (6) and (24) is derived.

**Remark 7**

For the case Γ_1_ = 0, the FDI (24) reduces to

(29) T1+Re{(T1Δ1+jωΛ)χ(jω)+ω2[I+Γ2χ(jω)]∗×T2[I+Δ2χ(jω)]}>0, ω∈ℝ,

which corresponds to the FDI as given in Theorem 1.15.1 in [4]. However, the results there only aim at single-input and single-output Lur'e systems.

If the slope restrictions on *φ*(*σ*(*t*)) are removed, another absolute stability criterion is derived by choosing (5) as the Lyapunov function.

**Theorem 8**

System (1) is absolutely stable for all *φ*(*σ*(*t*)) satisfying (2) if the matrix *A* + *B*Γ_1_ C* is Hurwitzian and there exist diagonal matrices Λ, *T* ≥ 0, symmetric matrices *P*, *Q* > 0, such that the following LMI is feasible:

(30) $$\begin{array}{c} \left[\begin{array}{cc} A^{*}P+PA-C\Gamma_{1}T\Delta_{1}C^{*} & PB+\Omega_{12}\\ B^{*}P+\Omega_{12}^{*} & \Omega_{22} \end{array}\right]<0, \end{array}$$

where *Ω*_12_ = (1/2)A*CΛ + (1/2)*CT*(Γ_1_ + Δ_1_), *Ω*_22_ = (1/2)ΛC*B + (1/2)B*CΛ − *T*.

**Proof**

The proof is similar to that of Theorem 3.

**Remark 9**

Theorem 8 gives absolute stability conditions for sector restricted Lur'e systems. In fact, the slope restricted condition (3) plays an important role in improving the condition of absolute stability. The forthcoming example shows that Theorem 3 is less conservative than Theorem 8.

Similar to Theorem 3, an equivalent frequency-domain criterion to Theorem 8 can be given as follows.

**Theorem 10**

System (1) is absolutely stable for all *φ*(*σ*(*t*)) satisfying (2) if the matrix *A* + *B*Γ_1_ C* is Hurwitzian and there exist diagonal matrices Λ, *T* ≥ 0 such that the following FDI holds:

(31) Re{[I+Γ1χ(jω)]∗T[I+Δ1χ(jω)]+jωΛχ(jω)}>0,ω∈ℝ.

**Proof**

From the KYP lemma, (30) is equivalent to

(32) [(jωI−A)−1BI]∗[−CΓ1TΔ1C∗Ω12Ω12∗Ω22][(jωI−A)−1BI]<0,∀ω∈ℝ.

The equivalence between (30) and (31) is derived from *χ*(*jω*) = C*(*A*−*jωI*)^−1^ *B* and C*A(*A*−*jωI*)^−1^ *B* = C*B + *jω* *χ*(*jω*).

**Remark 11**

Theorem 10 includes two particular cases. For the case Λ = 0, (31) is reduced to

(33) Re{(I+Γ1χ(jω))∗T(I+Δ1χ(jω))}>0, ω∈ℝ.

Correspondingly, Theorem 10 is in the form of the circle criterion. For the case Γ_1_ = 0, (31) reduces to

(34) T+Re{(jωΛ+TΔ1)χ(jω)}>0, ω∈ℝ.

Theorem 10 has the same form as the Popov criterion.

## 4. Numerical Example

In this section, a numerical example is presented to illustrate the effectiveness of the proposed results.

Consider Chua's oscillator [25] with the following dimensionless equations

(35) x˙1(t)=α[x2(t)−x1(t)−f(x1(t))],x˙2(t)=x1(t)−x2(t)+x3(t),x˙3(t)=−βx2(t)−γx3(t),

where *f*(*x*_1_(*t*)) = *m*_1_ *x*_1_(*t*) + (1/2)(*m*_0_ − *m*_1_)(|*x*_1_(*t*) + 1| − |*x*_1_(*t*) − 1|), *α*, *β*, *γ*, *m*_0_, and *m*_1_ are numbers. System (35) can be reformulated in the form of (1) with $x(t)=\left[\begin{array}{c} x_{1}(t)\\ x_{2}(t)\\ x_{3}(t) \end{array}\right]$, $A=\left[\begin{array}{ccc} -\alpha & \alpha & 0\\ 1 & -1 & 1\\ 0 & -\beta & -\gamma \end{array}\right]$, $B=\left[\begin{array}{c} -\alpha \\ 0\\ 0 \end{array}\right]$, $C={\left[\begin{array}{ccc} 1 & 0 & 0 \end{array}\right]}^{*}$, *σ*(*t*) = *x*_1_(*t*), and *φ*(*σ*(*t*)) = *m*_1_ *σ*(*t*) + (1/2)(*m*_0_ − *m*_1_)(|*σ*(*t*) + 1| − |*σ*(*t*) − 1|). The nonlinearity *φ*(*σ*(*t*)) satisfies

(36) $$\begin{array}{c} \min\left\{m_{0},m_{1}\right\}\sigma {\left(t\right)}^{2}\leq \varphi \left(\sigma \left(t\right)\right)\sigma \left(t\right)\leq \max\left\{m_{0},m_{1}\right\}\sigma {\left(t\right)}^{2},\\ \min\left\{m_{0},m_{1}\right\}\leq \frac{d\varphi \left(\sigma \left(t\right)\right)}{d\sigma \left(t\right)}\leq \max\left\{m_{0},m_{1}\right\}. \end{array}$$

Thus, Γ_1_ = Γ_2_ = min⁡{*m*_0_, *m*_1_} and Δ_1_ = Δ_2_ = max⁡{*m*_0_, *m*_1_}.

**Figure 1:**
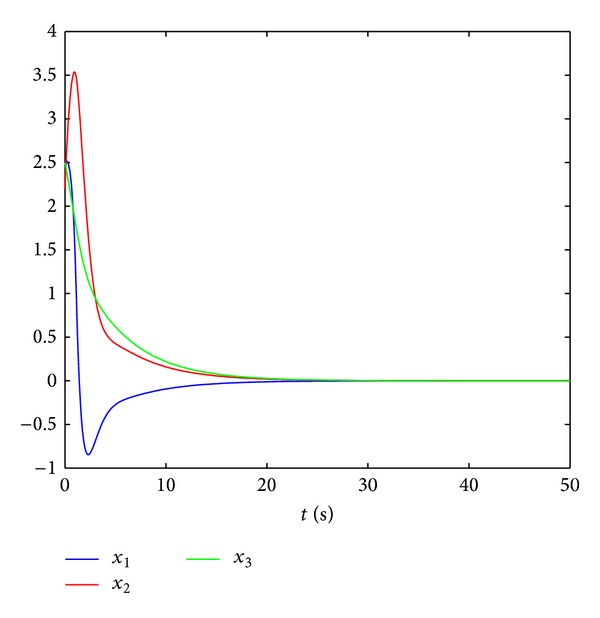
The states of system (35).

When *α* = −0.8018, *β* = 0.136, *γ* = 0.1097, and *m*_0_ = −2.96 are taken, system (35) is absolutely stable for *m*_1_ ≤ 2.009 by applying Theorem 3. However, we derive that system (35) is absolutely stable for *m*_1_ ≤ 1.81 and *m*_1_ ≤ 1.51, respectively, by Theorem 8 and the Popov criterion. This shows that Theorem 3 is an improvement with respect to Theorem 8 and the Popov criterion, and the slope restrictions could improve the absolute stability condition. The states of system (35) with *m*_1_ = 2 at the initial value ${\left[\begin{array}{ccc} 2.5 & 2.2 & 2.5 \end{array}\right]}^{*}$ are given in Figure 1, from which it is illustrated that system (35) is absolutely stable.

## 5. Conclusion

We have proposed new absolute stability criteria for Lur'e systems with sector and slope restricted nonlinearities from time-domain and frequency-domain points of view. The slope restrictions on nonlinearities improve the absolute stability conditions. We have shown that the criteria are less conservative than some existing results.
